# Durable Immunity to Ricin Toxin Elicited by a Thermostable, Lyophilized Subunit Vaccine

**DOI:** 10.1128/mSphere.00750-21

**Published:** 2021-11-03

**Authors:** Hayley Novak, Jennifer Doering, Dylan Ehrbar, Oreola Donini, Nicholas J. Mantis

**Affiliations:** a Department of Biomedical Sciences, University at Albany, Albany, New York, USA; b Division of Infectious Disease, Wadsworth Center, New York State Department of Health, Albany, New York, USA; c Soligenix, Inc., Princeton, New Jersey, USA; University of Florida

**Keywords:** vaccine, toxin, biodefense, mouse

## Abstract

The development of vaccines against biothreat toxins like ricin (RT) is considered an integral component of the U.S. national security efforts. RiVax is a thermostable, lyophilized RT subunit vaccine adsorbed to aluminum salt adjuvant intended for use by military personnel and first responders. Phase 1 studies indicated that RiVax is safe and immunogenic, while a three-dose intramuscular vaccination regimen in nonhuman primates elicited protection against lethal dose RT challenge by aerosol. Here, we investigated, in a mouse model, the durability of RiVax-induced antibody responses and corresponding immunity to lethal dose RT challenge. Groups of mice were subcutaneously administered 3 or 1 μg of RiVax on days 0 and 21 and challenged with 10× 50% lethal dose (LD_50_) RT by injection at six different intervals over the course of 12 months. Serum antibody titers and epitope-specific competition assays were determined prior to each challenge. We report that the two-dose, 3-μg regimen conferred near-complete protection against RT challenge on day 35 and complete protection thereafter (challenge days 65, 95, 125, 245, and 365). The two-dose, 3-μg regimen was superior to the 1-μg regimen as revealed by slight differences in survival and morbidity scores (e.g., hypoglycemia, weight loss) on challenge days 35 and 365. In separate experiments, a single 3-μg RiVax vaccination proved only marginally effective at eliciting protective immunity to RT, underscoring the necessity of a prime-boost regimen to achieve full and long-lasting protection against RT.

**IMPORTANCE** Ricin toxin (RT) is a notorious biothreat, as exposure to even trace amounts via injection or inhalation can induce organ failure and death within a matter of hours. In this study, we advance the preclinical testing of a candidate RT vaccine known as RiVax. RiVax is a recombinant nontoxic derivative of RT’s enzymatic subunit that has been evaluated for safety in phase I clinical trials and efficacy in a variety of animal models. We demonstrate that two doses of RiVax are sufficient to protect mice from lethal dose RT challenge for up to 1 year. We describe kinetics and other immune parameters of the antibody response to RiVax and discuss how these immune factors may translate to humans.

## INTRODUCTION

Ricin toxin (RT) is classified by the Centers for Disease Control and Prevention (CDC) as a biothreat toxin, alongside botulinum neurotoxin (BoNT), abrin, and Staphylococcus enterotoxin (SEB) (1). A recent NATO commission ranked RT as having a particularly high threat potential owing to its toxicity via inhalation, its ease of acquisition, and a lack of available countermeasures (2). RT is a product of the castor bean, Ricinus communis, which is propagated globally for its oils that are used in lubricants and cosmetics (3). In its mature form, RT is an ∼65-kDa cytotoxic glycoprotein consisting of two similarly sized subunits, RTA and RTB. RTB is a galactose/*N-*acetyl galactosamine (Gal/GalNAc)-specific lectin that facilitates RT entry into all mammalian cell types, including alveolar macrophages and airway epithelial cells (4). Once inside a cell, RTA is a highly efficient ribosome-inactivating protein (RIP) that triggers cell death and inflammation within a matter of hours (5–8). When inhaled, RT evokes a form of respiratory distress characterized by neutrophilic infiltration, intra-alveolar edema, accumulation of proinflammatory cytokines like interleukin 1 (IL-1) and IL-6 in bronchoalveolar lavage (BAL) fluids, and fibrinous exudate (9, 10). Monoclonal antibody (MAb) intervention studies in nonhuman primates indicate that the “point of no return” following an RT aerosol exposure is in the order of hours (11).

One of the most advanced candidate RT vaccines is RiVax, a recombinant, nontoxic derivative of RTA (12–14). RiVax and RiVax adsorbed to Alhydrogel are safe and immunogenic in healthy adults (15, 16). We have reported that RiVax is stable for at least 12 months at 40°C without loss of potency when formulated as a dry, glassy solid containing colloidal aluminum hydroxide adjuvant with trehalose as a stabilizing excipient (17, 18). In nonhuman primates, the thermostable RiVax formulation (100 μg) administered intramuscularly three times at monthly intervals stimulated immunity to subsequent lethal dose RT aerosol challenge (19). Protection correlates with the onset of RT-specific endpoint titers, as well as epitope-specific serum antibody responses revealed through a competition enzyme-linked immunosorbent assay (ELISA) known as EPICC (Ehrbar, C. Roy, G. Van Slyke, E. Vitetta, and N. Mantis, unpublished data) (19, 20).

In this report, we sought to further advance the preclinical analysis of the thermostable formulation of RiVax by examining the durability of protective immunity and RT-specific antibody responses induced following vaccination in mice. We were particularly interested in a prime-boost regimen in light of recent dose-response studies performed in Rhesus macaques (D. Ehrbar et al., unpublished).

## RESULTS

To assess the durability of RiVax-mediated immunity, 12 groups of mice were vaccinated subcutaneously (s.c.) with RiVax (3 μg; *n* = 12 per group) or vehicle (phosphate-buffered saline [PBS]) only (*n* = 6 per group) on days 0 and 21. Groups of mice were challenged with 10× 50% lethal dose (LD_50_) RT by i.p. injection on days 35, 65, 95, 125, 245, and 365 (Table 1). Two additional groups of mice were vaccinated s.c. with a low dose (1 μg) of RiVax on days 0 and 21 and challenged with RT on days 35 and 365 (Table 1). Serum samples were collected from animals 5 days prior to RT challenge and were assessed for RT-specific serum IgG endpoint titers by ELISA. The serum samples were also evaluated using an epitope-specific competition ELISA known as EPICC (20). As indicators of morbidity, we monitored animal weight loss and onset of hypoglycemia (21–23). A reference lot of RiVax was used for all experiments (18). The vaccine was reconstituted with sterile water for injection (WFI) immediately prior to use. It should be noted that, at all time points, groups of mice that were sham vaccinated succumbed to RT intoxication within 3 days (Table 1; Fig. S1 in the supplemental material).

**TABLE 1:** Response elicited by RiVax prime-boost vaccination regimen followed by i.p. RT challenge

Group	No. of mice	RiVax dose (μg)	RT (day)^*a*^	REPT^*b*^	% Inhibition (±SD)^*c*^ of:		Survival (no. [%])^*d*^
					R70	SyH7	
1	12	3.0	35	5.29	53.73 (23.56)	28.43 (16.3)	11/12 (92)
2	12	3.0	65	4.85	81.63 (15.66)	40.52 (26.96)	12/12 (100)
3	12	3.0	95	5.17	79.51 (14.95)	65.98 (16.13)	12/12 (100)
4	12	3.0	125	5.34	82.2 (17.78)	76.17 (11.37)	12/12 (100)
5	12	3.0	245	4.92	86.6 (13.98)	61.3 (17.43)	12/12 (100)
6	12	3.0	365	4.92	93.79 (2.99)	75.86 (12.52)	12/12 (100)
7	12	1.0	35	4.64	36.22 (23.19)	20.34 (18.6)	9/12 (75)
8	12	1.0	365	4.52	91.89 (6.39)	69.75 (18.71)	11/12 (92)
9	6		35	0	11.15 (1.94)	6.24 (2.8)	0/6 (0)
10	6		65	0	4.78 (1.77)	4.34 (3.13)	0/6 (0)
11	6		95	0	13.82 (10.15)	18.05 (4.15)	0/6 (0)
12	6		125	0	6.84 (6.06)	21.2 (2.2)	0/6 (0)
13	6		245	0	12.29 (6.15)	16.96 (3.5)	0/6 (0)
14	6		365	0	14.87 (5.02)	22.79 (2.27)	0/6 (0)

a RT challenge day.

b Log_10_-transformed reciprocal endpoint titers (REPT).

c EPICC inhibition values, as described in Materials and Methods.

d No. of survivors/total mice per group over course of 7 days post-RT challenge. The fates of control groups of mice are shown in Fig. S1 in the supplemental material.

10.1128/mSphere.00750-21.1 FIG S1 Fate of control mice (unvaccinated) following RT challenge. The control mice as described in Table 1 (corresponding to groups 9 to 14) were challenged with RT on the days indicated in panel A. (A) Kaplan-Meier survival curves; (B) percent change in body weight; (C) change in blood glucose levels. See Materials and Methods for details. Central lines represent group means, while error bars represent standard deviations. Download FIG S1, PDF file, 0.04 MB. Copyright © 2021 Novak et al. 2021 Novak et al. https://creativecommons.org/licenses/by/4.0/ This content is distributed under the terms of the Creative Commons Attribution 4.0 International license.

**FIG 1:**
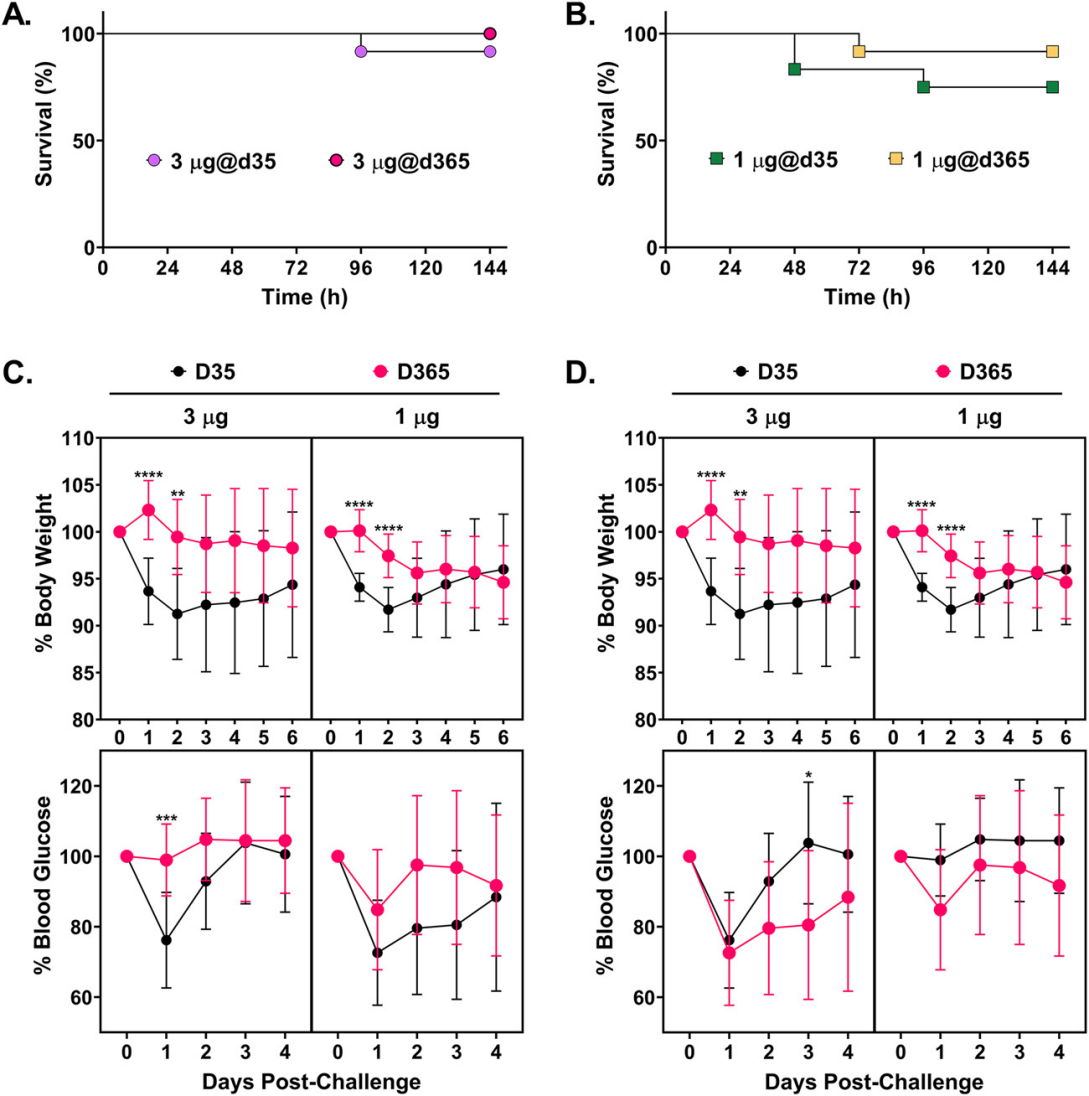
Durability of RiVax-induced immunity to ricin toxin (RT) over a period of 1 year. Groups of BALB/c mice were vaccinated with 1 μg or 3 μg RiVax on days 0 and 21 and then challenged with 10× LD_50_ at time points over the course of 1 year, as reported in Table 1. Kaplan-Meier curves of groups of mice vaccinated with 1 μg (A) or 3 μg (B) RiVax and challenged with RT on day 35 or 365. Survival was monitored over a period of 7 days. (C and D) Body weight and blood glucose levels following RT challenge, plotted as a function of RiVax dose and day of challenge. The fate of the control (unvaccinated) mice is reported in Fig. S1 in the supplemental material. The body weights and blood glucose levels of vaccinated mice challenged at interim time points are reported in Fig. S2. Shown are mean values ± SD. Significance was determined using two-way ANOVAs followed by Šidák’s multiple-comparison tests. *, *P* ≤ 0.05; **, *P* < 0.01; ***, *P* < 0.001; ****, *P* < 0.0001.

The group of mice that received 3 μg of RiVax and challenged with RT on day 35 had a 92% (11/12) survival rate (Table 1; Fig. 1). At time points thereafter, the groups of mice that received high-dose (3 μg) RiVax exhibited complete survival (Table 1; Fig. 1). From the standpoint of morbidity, mice that were vaccinated with 3 μg of RiVax and challenged on day 35 experienced significant weight loss and hypoglycemia in the days immediately following toxin exposure, whereas mice challenged on day 365 (or any other day besides day 35) did not (Fig. 1; Fig. S2). Mice that received the low dose of RiVax (1 μg) exhibited 75% (9/12) survival upon RT challenge on day 35 and 92% (11/12) survival on day 365. The low-dose vaccinated mice that survived RT challenge did not experience more severe weight loss or hypoglycemia than their counterparts that received high-dose RiVax (Fig. 1). Collectively, these data demonstrate that a prime-boost regimen with RiVax (3 μg) elicits a protective immune response to RT that matures between days 35 and 60 and persists for at least 12 months.

10.1128/mSphere.00750-21.2 FIG S2 Morbidity-associated RT challenge in RiVax-vaccinated mice over the course of a year. Groups of mice were vaccinated with 3 mcg RiVax on days 0 and 21 and then challenged with RT by injection on days 35 (A), 65 (B), 95 (C), 125 (D), 245 (E), and 365 (F) as described in Table 1. Body weight (top panels) measured daily for 6 days and blood glucose levels (lower panels) measured daily for 4 days after RT challenge. Central lines represent group means, while error bars represent standard deviations. Download FIG S2, PDF file, 0.05 MB. Copyright © 2021 Novak et al. 2021 Novak et al. https://creativecommons.org/licenses/by/4.0/ This content is distributed under the terms of the Creative Commons Attribution 4.0 International license.

**FIG 2:**
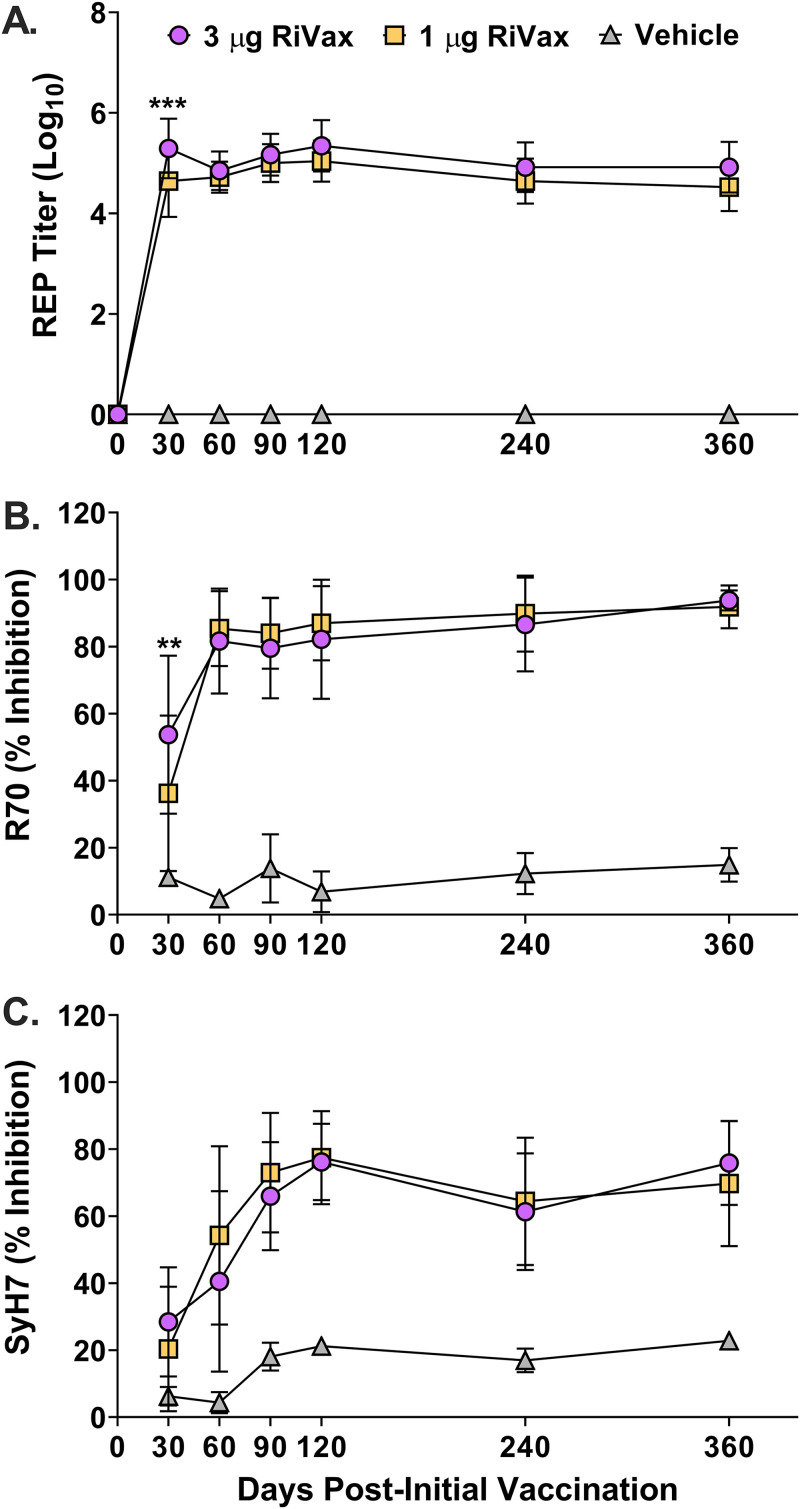
Durability of RT- and epitope-specific antibody in responses of mice following RiVax vaccination. Graphical representations of log_10_-transformed RT-specific serum IgG reciprocal endpoint titers (A) and EPICC inhibition values from mice vaccinated with 1 μg or 3 μg RiVax on days 0 and 21 (B and C), as reported in Table 1. Sera were collected on the experimental days indicated on the *x* axis. In all cases, mean values ± SD are shown. Mixed-effects analyses followed by Šidák’s multiple-comparison tests were used to determine significant differences between groups. *, *P* ≤ 0.05; **, *P* < 0.01; ***, *P* < 0.001; ****, *P* < 0.0001.

In light of these results, we evaluated the kinetics of RT-specific serum IgG endpoint titers in the low- and high-dose RiVax-vaccinated mice (Fig. 2). On day 30, there was an ∼5-fold significant difference in endpoint titers between mice that received 1 μg (4.442 log_10_-transformed reciprocal endpoint titers [REPT]) and 3 μg (5.118 REPT) (Table 1; Fig. 2). However, on day 60 and beyond, the REPTs were similar (Table 1; Fig. 2), indicating the high dose of RiVax accelerated the onset of RT-specific antibody titers by day 30, but thereafter, antibody levels were equivalent and remained steady for the duration of the 12-month study.

We next used EPICC to assess differences in epitope-specific antibody responses over time and between the low and high RiVax doses. EPICC is a modified-capture ELISA in which microtiter plates are coated with MAbs and then probed with soluble biotinylated RT in the absence or presence of a competitor antiserum (20). R70 and SyH7 are toxin-neutralizing MAbs that recognize two spatially distinct immunodominant epitopes on RTA (24). A reduction in captured biotinylated RT by R70 and/or SyH7 is inversely proportional to the number of competitor antibodies present in the query antisera (20). Furthermore, we have demonstrated that EPICC inhibition values serve as a correlate of vaccine-induced immunity to RT in mice (20).

We found that R70 EPICC inhibition values were detectable by day 30 in the sera of mice that received low- and high-dose RiVax, plateaued at day 60, and persisted for the remainder of the study (Fig. 2B). In the 3-μg RiVax group, R70 EPICC inhibition values were significant compared to the 1-μg RiVax group on day 30 but were essentially identical thereafter (Fig. 2B). SyH7 EPICC inhibition levels for both vaccination groups were low on day 30 but rose until they plateaued on day 120, reflecting a delay in maturation of the SyH7 response (Fig. 2C). The EPICC inhibition values persisted for at least 360 days and corresponded with overall immunity to RT challenge.

### Immunity to lethal dose RT challenge elicited by a single RiVax vaccination.

It has become apparent in the COVID-19 pandemic that, in public health emergencies, it may be necessary to implement single-dose vaccinations when supplies of vaccines are limited. To investigate the benefit afforded by a single dose of RiVax (3 μg), a cohort of mice were vaccinated s.c. with 3 μg of RiVax on day 0. Groups of mice (*n* = 12) were then challenged with 10× LD_50_ RT on days 35, 65, and 95. As noted above, we monitored mice for weight loss and hypoglycemia as indicators of toxin-induced morbidity. In addition, serum samples prior to toxin challenge were evaluated by RT-specific ELISA and EPICC.

**FIG 3:**
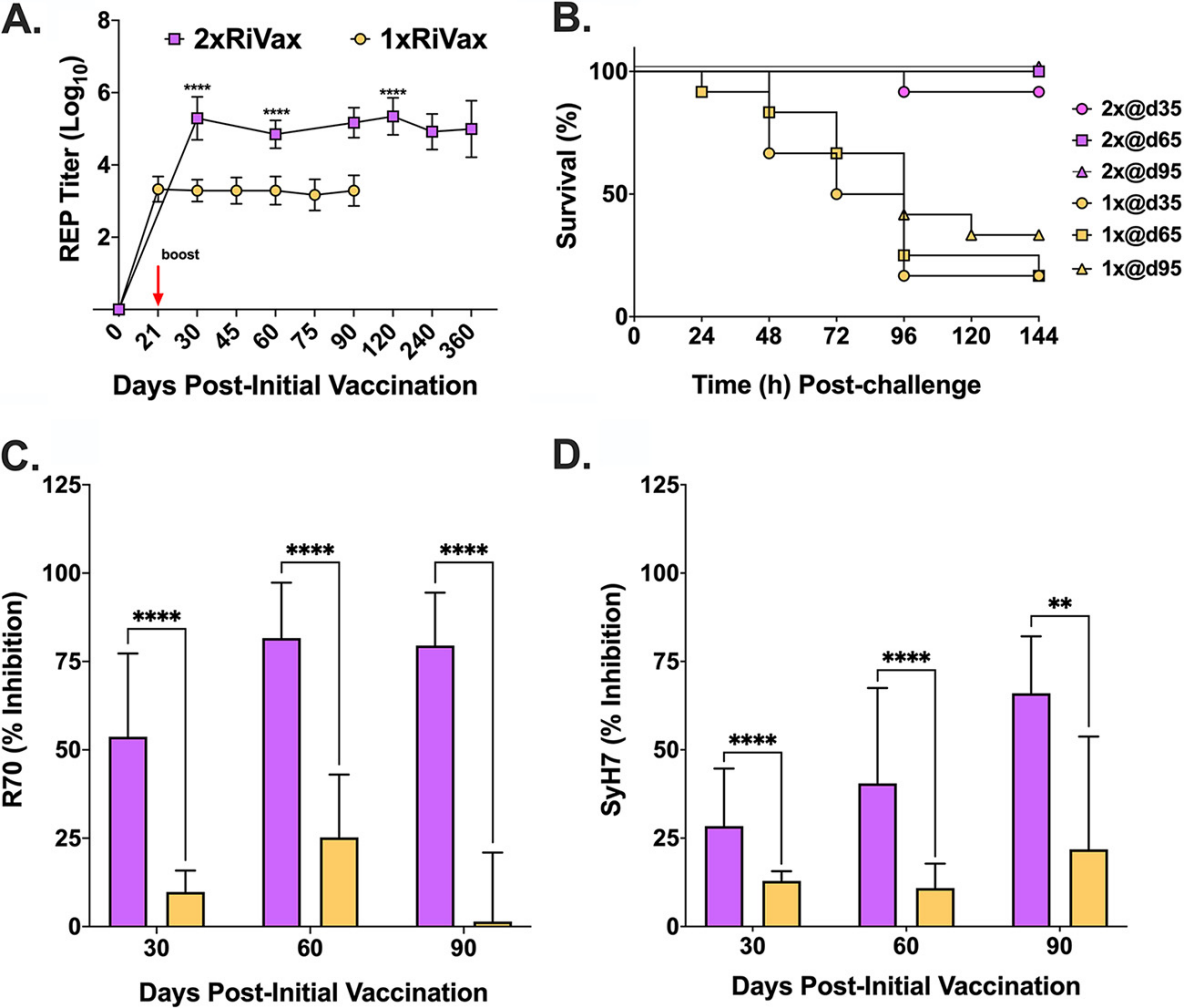
Comparison of single- and two-dose RiVax vaccinations on immunity to ricin toxin. Graphical representations of results presented in Table 2. (A) Log_10_-transformed RT-specific serum IgG reciprocal endpoint (REP) titers from mice vaccinated with 3 μg RiVax on day 0 (1 × RiVax; gold symbols) or on days 0 and 21 (2 × RiVax; purple symbols). (B) Kaplan-Meier plots of groups of RiVax-vaccinated mice challenged on study days 35, 65, and 95. (C and D) EPICC inhibition values from sera collected on study days 30, 60, or 90 from mice vaccinated with 3 μg RiVax once (gold) or twice (purple). A two-way ANOVA followed by a Šidák’s multiple-comparison test was used to determine significant differences in REPT between groups, while mixed-effects models followed by Šidák’s tests were used to examine differences in EPICC inhibition. The single mouse that died due to RT challenge on day 35 was excluded from this analysis. *, *P* ≤ 0.05; **, *P* < 0.01; ***, *P* < 0.001; ****, *P* < 0.0001.

The single-vaccination regimen proved vastly inferior to the two-dose regimen in terms of eliciting protective immunity to RT. Only 17% (2/12) of the mice that received the single vaccine survived RT challenge on days 35 and 65 (Table 2). On day 95, survival was just 33% (4/12), compared to 100% in the two-dose regimen (Table 2; Fig. 3). Of note, RT-specific serum IgG levels in the mice that received the single dose of RiVax were similar on days 30, 60, and 90, revealing the absence of any maturation of antibody titers beyond day 30 (Fig. 3). In fact, the single dose of RiVax elicited significantly lower RT-specific serum IgG titers across the board, according to a mixed-effects analysis with Šidák’s multiple-comparison test. Those same groups of mice had lower mean EPICC inhibition values than the mice that received two doses of RiVax (Fig. 3; Fig. S3). These data demonstrate that, at the dose tested, little benefit is afforded by a single vaccination with RiVax.

**TABLE 2:** Response elicited by single RiVax vaccination regimen

Group	No. of mice	RiVax dose (μg)	RT (day)^*a*^	REPT^*b*^	% Inhibition (±SD)^*c*^ of:		Survival (no. [%])^*d*^
					R70	SyH7	
1	12	3.0	35	3.289	9.80 (6.07)	12.92 (2.75)	2/12 (17)
2	12	3.0	65	3.289	25.24 (17.79)	10.87 (6.91)	2/12 (17)
3	12	3.0	95	3.289	1.45 (19.49)	21.82 (31.91)	4/12 (33)
							
4	6		35	0	5.98 (2.06)	14.6 (2.83)	0/6 (0)
5	6		65	0	13.23 (3.46)	8.74 (5.85)	0/6 (0)
6	6		95	0	−12.58 (3.83)	9.67 (26.41)	0/6 (0)

a RT challenge day.

b Log_10_-transformed reciprocal endpoint titers (REPT).

c EPICC inhibition values, as described in Materials and Methods and provided in Fig. S3.

d No. of survivors/total mice per group over course of 7 days post-RT challenge.

10.1128/mSphere.00750-21.3 FIG S3 REPT and EPICC inhibition values in survivors and decedents following single RiVax vaccination and RT challenge. Mice were vaccinated with a single dose of 3 μg of RiVax at day 0, as described in Table 2. Log_10_-transformed RT-specific reciprocal endpoint (REPT) titers and EPICC inhibition values were measured in sera collected 5 days before RT challenge, as described in Materials and Methods. Each symbol represents an individual mouse. Central lines represent group means, while error bars represent standard deviations. Download FIG S3, PDF file, 0.06 MB. Copyright © 2021 Novak et al. 2021 Novak et al. https://creativecommons.org/licenses/by/4.0/ This content is distributed under the terms of the Creative Commons Attribution 4.0 International license.

### Mucosal immunity to RT elicited by RiVax vaccination.

RT is particularly toxic when inhaled (10, 22). While numerous reports have indicated that parenteral (systemic) RiVax vaccination is sufficient to confer immunity against a pulmonary RT challenge, those studies have been done with three doses, not two (19, 22). Therefore, cohorts of mice (*n* = 12) were vaccinated s.c. with 0.3, 1, or 3 μg of RiVax on days 0 and 21. Mice were then challenged intranasally with a 10× LD_50_ of RT on days 35, 125, and 162. Serum samples were collected from the animals 5 days prior to all the challenge days (plus an additional collection on day 90) and then evaluated by ELISA and EPICC (Table 3).

**TABLE 3:** Response elicited by RiVax prime-boost vaccination regimen followed by i.n. challenge

Group	No. of mice	RiVax dose (μg)	RT (day)^*a*^	REPT^*b*^	% Inhibition (±SD)^*c*^ of:		Survival (no. [%])^*d*^
					R70	SyH7	
1	12	0.3	35	3.130	15.09 (16.80)	6.25 (8.98)	0/12 (0%)
2	12	1	35	4.230	38.78 (25.98)	20.09 (17.0)	4/12 (33%)
3	12	3	35	4.734	56.28 (25.05)	28.4 (21.76)	7/12 (58%)
							
4	12	0.3	125	3.150	39.25 (31.49)	24.43 (20.83)	0/12 (0%)
5	12	1	125	4.144	86.40 (15.77)	67.12 (17.78)	9/12 (75%)
6	12	3	125	4.363	82.85 (18.06)	60.75 (20.58)	10/12 (83%)
							
7	12	0.3	162	3.766	35.34 (39.44)	33.62 (26.6)	1/12 (8.3%)
8	12	1	162	4.124	60.43 (33.34)	64.23 (19.24)	8/12 (67%)
9	12	3	162	4.403	70.57 (24.26)	62.6 (21.73)	9/12 (75%)
							
10	6	NA	35	0	6.23 (3.34)	8.62 (2.76)	0/6 (0%)
11	6	NA	125	0	3.66 (3.33)	13.86 (3.27)	0/6 (0%)
12	6	NA	162	0	6.04 (11.47)	5.07 (4.11)	0/6 (0%)

a RT challenge day.

b Log_10_-transformed reciprocal endpoint titers (REPT).

c EPICC inhibition values, as described in Materials and Methods.

d No. of survivors/total mice per group over course of 7 days post-RT challenge.

**FIG 4:**
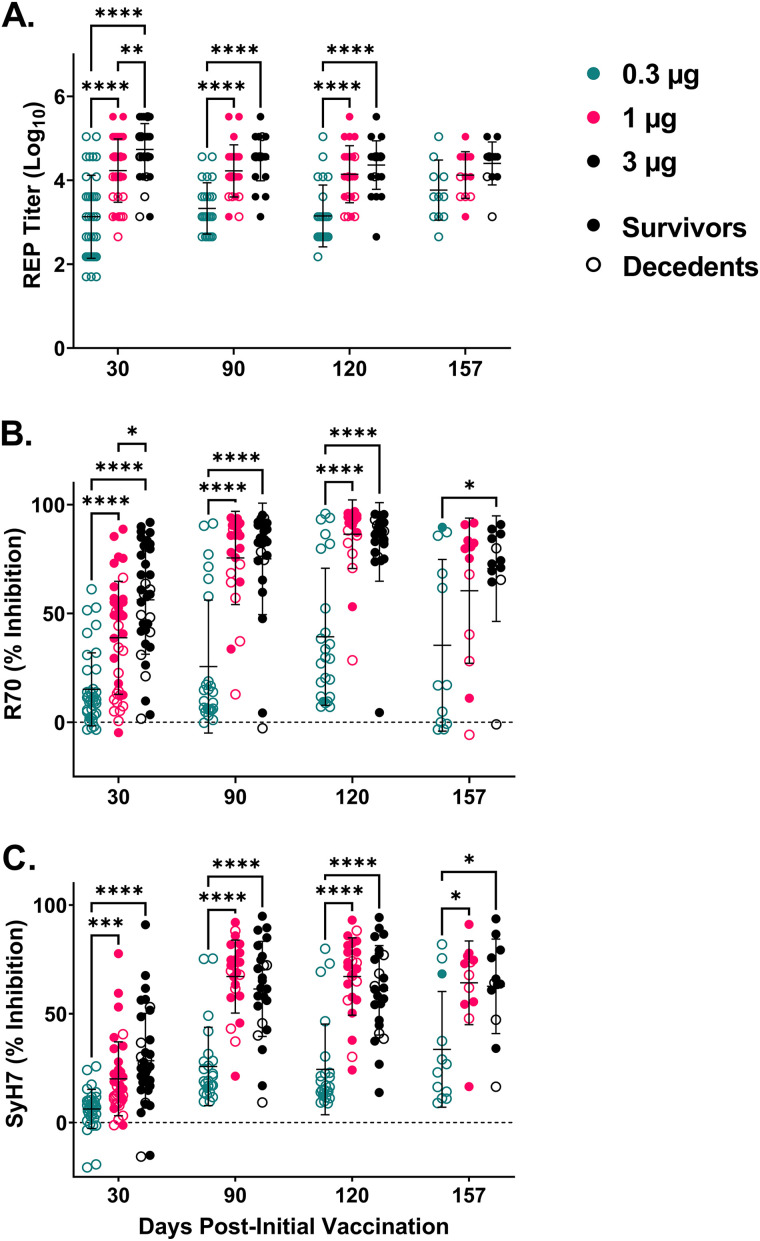
Serum antibody titers and EPICC inhibition values of R70 and SyH7 increased significantly with RiVax dose. Mice were vaccinated with 0.3, 1, or 3 μg of RiVax on days 0 and 21. REPT and EPICC inhibition values were determined for serum collected on days 30, 90, 120, and 157. (A) Reciprocal endpoint titers were significantly higher in the 3-μg group than the 0.3-μg group at the 30, 90, and 120 time points and were also significantly higher than the 1-μg group on day 30. (B) Similarly, R70 inhibition was significantly higher in the 3-μg than the 0.3-μg group at all time points and higher than the 1-μg group on day 30. Inhibition in the 1-μg group was significantly higher than the 0.3-μg group on days 30, 90, and 120. (C) Levels of SyH7 inhibition were also significantly higher in the 3- and 1-μg groups than the 0.3-μg group on all days. Mixed-effects analyses followed by Šidák’s multiple-comparison tests were used to determine significant differences between groups. *, *P* ≤ 0.05; **, *P* < 0.01; ***, *P* < 0.001; ****, *P* < 0.0001.

Overall, mice that were vaccinated with 0.3, 1, or 3 μg of RiVax on days 0 and 21 fared poorly when challenged with RT intranasally on day 35. Indeed, only a single mouse that received the low dose of RiVax (0.3 μg) survived RT challenge (Table 3). In mice that received 1 μg of RiVax, survival ranged from 33% on day 35 to 67% and 75% on days 125 and 162, respectively. Groups of mice that received 3 μg of RiVax had 58% (7/12) survival on day 35, 83% (10/12) on day 125, and 75% (9/12) on day 162 (Table 3). The kinetics of RT-specific antibody responses were similar to those observed previously in that there were both dose- and time-dependent components to the onset of RT-specific serum IgG levels (Table 3). The 3-μg group displayed the highest titers and were significantly different from the 0.3-μg group at all time points, except day 157 (Fig. 4A). R70 and SyH7 EPICC serum inhibition values were highest in the 3-μg RiVax group of mice (Fig. 4B and C). Comparing groups of vaccinated mice by the same regimen but challenged by the intraperitoneal (i.p.) versus intranasal (i.n.) routes suggests a higher serum IgG threshold associated with protection against 10× LD_50_ RT mucosal challenge.

## DISCUSSION

The development of medical countermeasures against select agents and toxins remains a research priority in the United States, even as the country is struggling to contain a resurgence of COVID-19. In this report, we advanced preclinical studies of RiVax, a candidate RT subunit vaccine intended for use in military and high-risk civilian populations (25). We found that a parenteral prime-boost RiVax vaccination regimen in mice was sufficient to stimulate protective immunity to RT for at least 12 months. RT-specific serum IgG levels peaked between days 30 and 60 and then persisted for the duration of the year-long study. Epitope-specific antibody responses, which are known to correlate with protective immunity to RT in mice, were equally durable (20). However, mice that received only a single dose of RiVax mounted significantly lower RT-specific serum IgG levels compared to their two-dose counterparts and fared poorly upon RT challenge, likely due to insufficient maturation of the toxin-neutralizing antibody response. In summary, we conclude that a prime-boost regimen of RiVax adjuvanted with Alhydrogel is both sufficient and necessary to elicit sustained immunity to parenteral RT challenge.

While the primary endpoint for assessing RiVax efficacy in this study was survival, secondary measures of toxin-induced morbidity, namely, weight loss and hypoglycemia, provided insight into relative degrees of vaccine-induced immunity not evident from simple Kaplan-Meier plots (12, 21, 22, 26). For example, mice vaccinated with 3 μg of RiVax on days 0 and 21, then challenged on day 35, experienced significantly greater weight loss and hypoglycemia in the days immediately following toxin challenge than did an identically vaccinated group of mice challenged on day 365. This observation is consistent with a maturation of the RT antibody response over a period of >60 days, as has been noted previously in mice (27) and even nonhuman primates (19). In Rhesus macaques, for example, toxin-neutralizing antibody titers elicited following RiVax vaccination peaked several months after RT-specific IgG levels were maximal (19).

Another metric used in this study to interrogate the RT-specific antibody response following RiVax vaccination was EPICC, a competition ELISA with two toxin-neutralizing MAbs, R70 and SyH7, that target spatially distinct epitopes on RiVax (24). In a recent study, we demonstrated in a large cohort of RiVax-vaccinated mice that EPICC values combined with RT-specific endpoint titers in a multivariate model resulted in greater performance at predicting survival following lethal dose RT challenge than any one of the independent variables alone (20). EPICC inhibition values also serve as correlates of RiVax-induced protection in NHPs (C. Roy, D. Ehrbar, G. Van Slyke, E. Vitetta, O. Donini, N. Mantis, unpublished data). While survival predictions were not necessarily applicable to the current study given the overall high survival rate, the kinetics of R70 and SyH7 EPICC inhibition values can be considered readouts of RT-specific antibody maturation. For example, R70 EPICC inhibition values following two-dose RiVax vaccination peaked on day 60, even though RT-specific endpoint titers peaked on day 30. In this respect, R70 EPICC inhibition values corresponded to the time point where full protection against RT challenge was achieved. SyH7 EPICC inhibition values peaked even later (day 120), possibly reflecting the subdominant nature of SyH7’s epitope, which is located on the backside of RTA relative to the active site (24). While we have not yet defined an absolute EPICC inhibition “threshold” associated with protective immunity to RT, the cumulative differences in R70 and SyH7 EPICC inhibition values between groups of vaccinated mice that survived RT challenge and those that did not are often stark, as exemplified in Fig. 4.

Results from the RiVax studies involving parenteral vaccination followed by intranasal RT challenge reinforces the notion that the threshold of protection against RT respiratory exposure is greater than systemic exposure. In other words, higher levels of RT-specific serum IgG titers are required to protect mice against an intranasal (or aerosol) RT challenge compared to a parenteral (intraperitoneal) challenge. From passive MAb immunization studies in mice, we estimated that the threshold for immunity against intranasal RT challenge is about ∼8- to 10-fold greater than what was required to protect mice against the same amount of toxin given by intraperitoneal injection (28). We speculate that the higher serum IgG levels are due to issues related to lung bioavailability, as noted by others (29). Specifically, high serum IgG levels may be necessary to ensure sufficient transudation and/or transport of antibodies into the airways where they can intercept and neutralize RT before the toxin is internalized by alveolar macrophages and lung epithelial cells (28). The issue of serum IgG delivery into the lung has come to the forefront recently when considering SARS-CoV-2 vaccine efficacy and MAb therapy aimed at dampening viral replication and inflammation (30, 31). Of note, previous studies with aerosol exposure in Rhesus macaques have demonstrated that it is entirely feasible to obtain these higher levels of protection (19).

An investigation into the benefits of combining RiVax with an additional adjuvant may be warranted in light of emerging work on SARS-CoV-2 RBD subunit vaccines. Of particular note, there has been the success of aluminum salts combined with CpG in stimulating SARS CoV-2-neutralizing antibody titers in mice (32, 33). The aluminum salts plus CpG combination also stimulated unique cytokine and chemokine profiles in human peripheral blood mononuclear cells (PBMCs) (33). The RiVax antigen is compatible with other adjuvants such as LT-II (34, 35) and alpha-galactosyl ceramide (36), but, to our knowledge, RiVax adsorbed to aluminum hydroxide has not itself been combined with other adjuvants. Moreover, whether such adjuvants would be compatible with the RiVax lyophilization protocol would also need to be investigated (17), although success with a similar lyophilized formulation and a nano-emulsion adjuvant (CoVaccine HT) has been demonstrated (37).

## MATERIALS AND METHODS

### Ricin toxin and RiVax vaccine formulations.

Ricin toxin (Ricinus communis agglutinin II; RT) and RTA were purchased from Vector Laboratories (Burlingame, CA). RT was purchased without azide, and sterility of the preparation was confirmed prior to use in animal studies. A single lot of lyophilized RiVax formulated as 10 mM histidine, 8% (wt/vol) trehalose, 200 μg/ml RiVax, and 0.85 mg/ml aluminum, pH 6.5) was used for all experiments (19). RiVax was reconstituted with sterile water for injection (1 ml) immediately prior to use.

### Mouse vaccination studies.

Mouse studies were conducted under strict compliance with the Wadsworth Center’s Institutional Animal Care and Use Committee (IACUC). Female BALB/c mice aged 6 to 8 weeks were obtained from Taconic Biosciences (East Greenbush, NY) and housed under conventional, specific-pathogen-free conditions. Mice (*n* = 12 per experimental group; *n* = 6 to 8 for control groups) were vaccinated via the subcutaneous (s.c.) route on days 0 and 21. The vaccines were administered to mice on days 0 and 21 in 50-μl final volumes. Blood was collected from mice via the submandibular vein on days indicated in the figures.

### RT challenge.

Animal experiments were conducted in compliance with the Wadsworth Center’s IACUC. The Wadsworth Center is an AAALAC-accredited institution. Mice were challenged by intraperitoneal (i.p.) injection or intranasal instillation (under isoflurane anesthesia) with the equivalent of 10× LD_50_ (100 μg/kg) RT. Challenge doses >10× LD_50_ are not permitted under our IACUC protocol. Survival was monitored over a 7-day period. Blood glucose levels were measured for 4 days postchallenge and body weight daily until day 7. As per protocol, mice were euthanized when they became overtly moribund, experienced weight loss ≥ 20% of prechallenge weight, and/or became hypoglycemic (<25 mg/dl) following i.p. challenge or (<49 mg/dl) for i.n. challenge.

### ELISA.

Nunc Maxisorb F96 microtiter plates (Thermo Fisher Scientific, Pittsburgh, PA) were coated overnight at 4°C with RT (1 μg/well in PBS), washed with PBS with 0.05% (vol/vol) Tween 20 (PBS-T), and then blocked for 2 h with PBS-Tween (PBST) containing 2% (vol/vol) goat serum (Gibco, MD, USA). Threefold serial dilutions of serum (starting at 1:50) were then applied to plate for 1 h at ambient temperature, washed, and detected with horseradish peroxidase (HRP)-conjugated goat anti-mouse IgG (SouthernBiotech, Birmingham, AL). The ELISA plates were developed using SureBlue 3,3′,5,5′-tetramethylbenzidine (TMB; Kirkegaard & Perry Labs, Gaithersburg, MD) and analyzed using a SpectraMax 250 spectrophotometer equipped with Softmax Pro 5.4.5 software (Molecular Devices, Sunnyvale, CA). The endpoint titer was defined as the minimal dilution whose absorbance (450 nm) was >3 times background, with background being defined as the average absorbance produced by wells with buffer alone. Endpoint titers were log_10_ transformed before further analysis. Vero cell cytotoxicity assays were performed as previously described (38, 39).

### EPICC analysis.

Epitope profiling immunocapture competition (EPICC) assays were conducted as described (20). Clear, flat-bottom Immulon 4HBX 96-well plates (Thermo Fisher Scientific, Pittsburgh, PA) were coated overnight with a capture anti-RTA MAb (1 μg/ml). The following day, the plates were blocked with 2% goat serum, washed, and then overlaid with a mixture of biotin-tagged RT and polyclonal antibody (pAb) mouse serum (1:25). The amount of biotin-RT used in the competition ELISA was equivalent to the 90% effective concentration (EC_90_) for each capture MAb (range, 100 to 150 ng/ml). After 1 h of incubation, plates were washed and developed with streptavidin-HRP (1:1,000; Thermo Fisher Scientific, Pittsburgh, PA) and TMB, as described above for ELISAs. The percent inhibition of RT binding to the capture MAb in the presence of pAb serum was calculated from the optical density (OD) values as follows: 1 − value OD_450_ value (biotin-RT + competitor MAb)/OD_450_ (biotin-RT without competitor MAb) × 100.

### Statistical analysis.

Statistical analyses were performed with GraphPad Prism v.8.01. Reciprocal endpoint titers were log transformed prior to statistical analysis. Two-way analyses of variance (ANOVAs) or mixed-effects models followed by Šidák’s multiple-comparison tests were performed to determine significant differences between reciprocal endpoint titers and EPICC inhibition levels, as described in the figure legends. Survival data were tested using the log rank Mantel-Cox test. In all cases, the significance threshold was set at a *P* value of <0.05.
