# Supplementary figures and images for: Durable Immunity to Ricin Toxin Elicited by a Thermostable, Lyophilized Subunit Vaccine
Source: mSphere. 2021 Nov 3;6(6):e00750-21. doi: 10.1128/mSphere.00750-21 (PMC8565519; doi:10.1128/mSphere.00750-21)

**Figure S1**

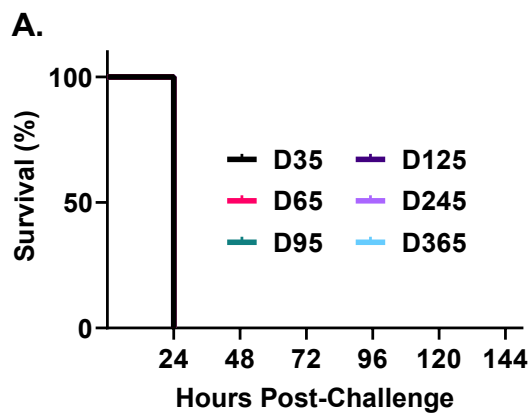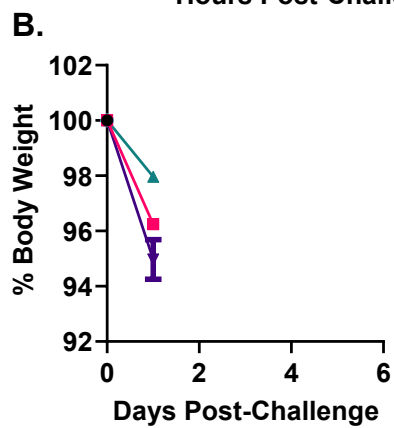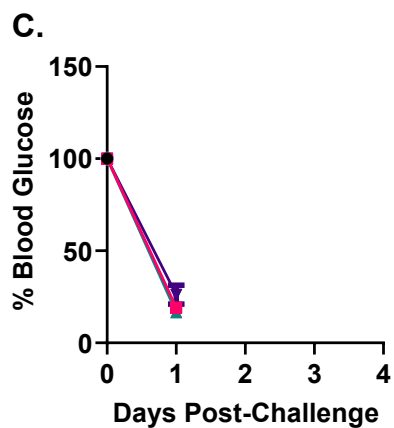

Supplement: FIG S1 [file msphere.00750-21-sf001.pdf]

**Figure S2**

**A. D35**

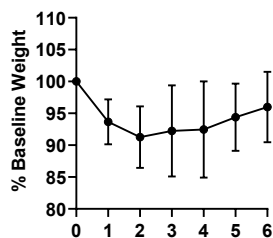

**B. D65**

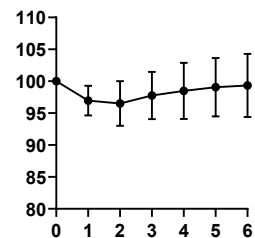

**C. D95**

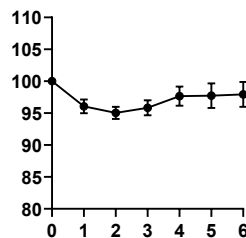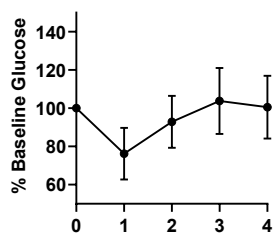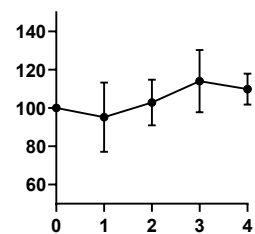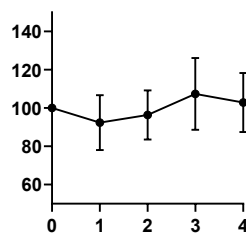

**D. D125**

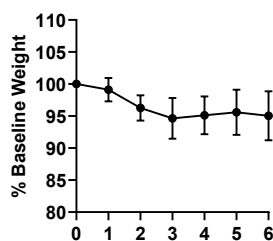

**E. D245**

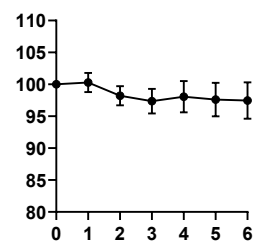

**F. D365**

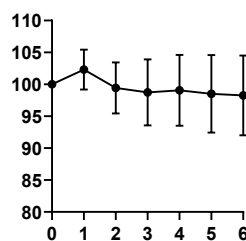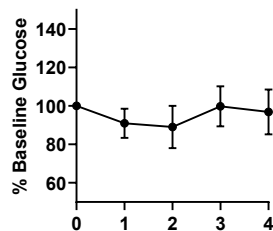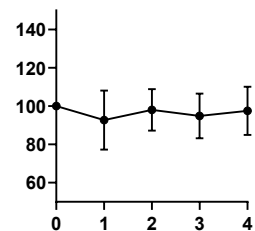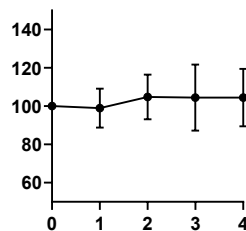

**Days Post-Challenge**

Supplement: FIG S2 [file msphere.00750-21-sf002.pdf]

Figure S3

A.

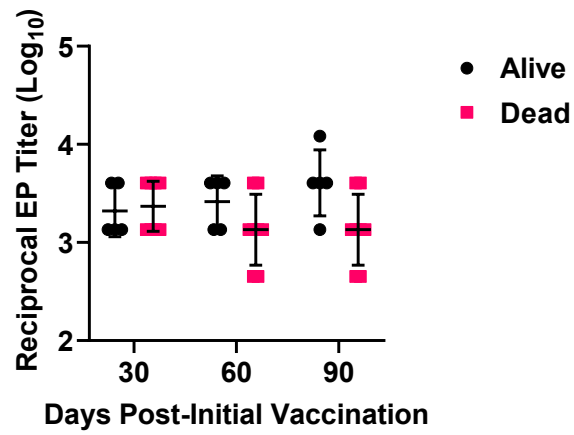

B.

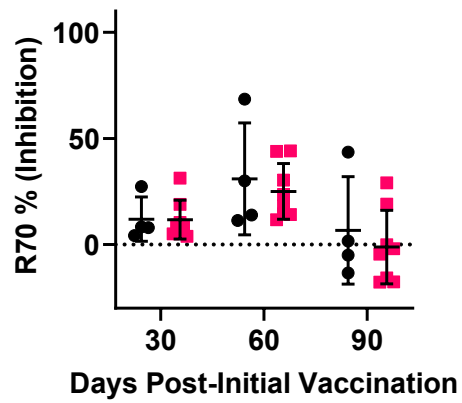

C.

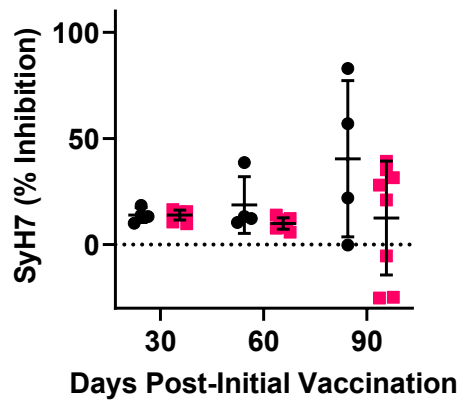

Supplement: FIG S3 [file msphere.00750-21-sf003.pdf]
